# Supplementary material for: Enhanced elastocaloric cooling beyond Clausius–Clapeyron limits
Source: Nat Commun. 2026 Apr 27;17:3747. doi: 10.1038/s41467-026-72172-7 (PMC13121465; doi:10.1038/s41467-026-72172-7)
Supplement: Supplementary file 1 — Supplementary information [file 41467_2026_72172_MOESM1_ESM.pdf]

Supplementary Information to

"Enhanced elastocaloric cooling beyond Clausius–Clapeyron limits"

Authors: Yuxin Song [1, 2], Sheng Xu [1, 2]*, Toshihiro Omori [2], Takuro Kawasaki [3], Yoshihisa Ishikawa [4], Ryoji Kiyanagi [3], Ryosuke Kainuma [2]

Affiliations:

[1] Frontier Research Institute for Interdisciplinary Sciences, Tohoku University, Aramaki aza Aoba 6-3, Sendai 980-8578, Japan.

[2] Department of Materials Science, Graduate School of Engineering, Tohoku University, Aoba-yama 6-6-02, Sendai 980-8579, Japan.

[3]J-PARC Center, Japan Atomic Energy Agency, Tokai, Ibaraki 319-1195, Japan.

[4] Neutron Science and Technology Center, Comprehensive Research Organization for Science and Society, Tokai, Ibaraki 319-1106, Japan

*Corresponding author. Email: xu.sheng.a8@tohoku.ac.jp (S. X.)

Contents:

Supplementary Discussion.

Supplementary Figures 1 to 7.

Supplementary Table 1.

Supplementary References.

Supplementary Discussion

1. <u>Phase characterization of as-made Ti-Al-Cr shape memory alloy</u>

Ti-Al-Cr shape memory alloys are characterized by the B2 parent phase unlike most Ti-based shape memory alloys [1]. Supplementary Fig. 1 displays the XRD profile of the Ti-Al-Cr alloy bulk sample after solid-solution treatment at 1473 K and subsequent aging at 473 K for 1 hour. A distinct B2 (100) superlattice reflection was observed, confirming the ordered B2 structure in accordance with our previous findings [1].

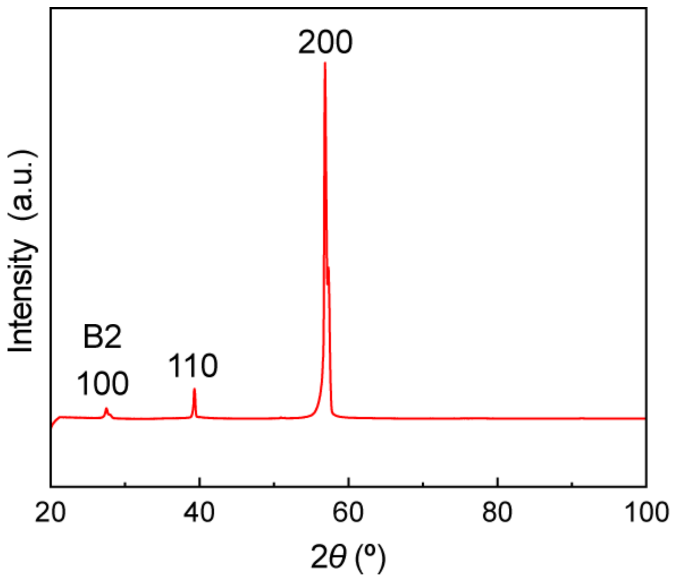

Supplementary Figure 1. XRD profile of as-made Ti-Al-Cr alloy.

2. <u>Superelastic training at room temperature</u>

The first 10 mechanical compressive cycles for Ti-Al-Cr single crystal were shown in Supplementary Fig. 2. This revealed general responses of superelasticity to mechanical cycling: the σ_{Ms} and transformation hysteresis decreases due to the superelastic training. The transformation stress became stable after such 10-cycle training.

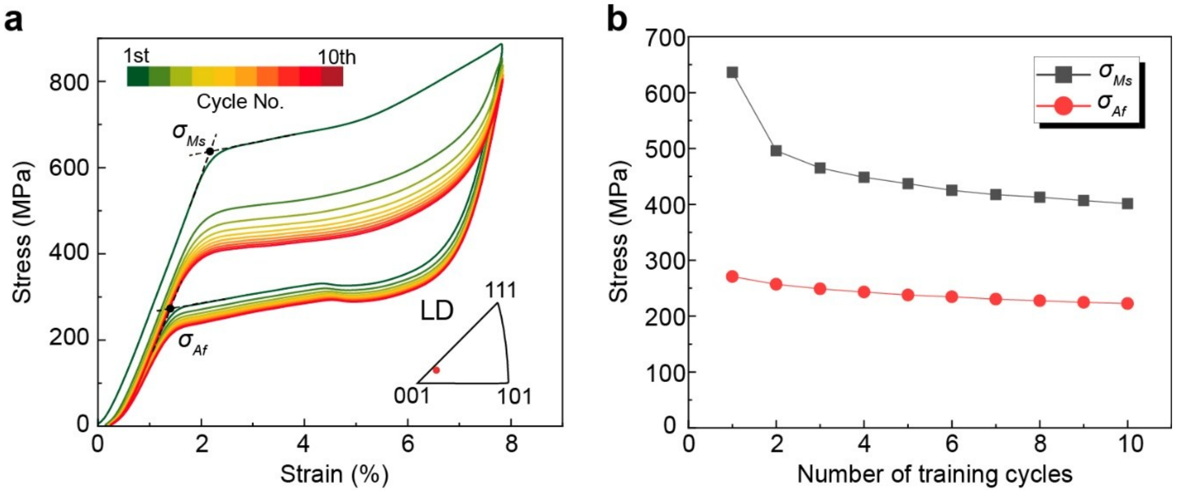

Supplementary Figure 2. Room-temperature cyclic mechanical tests.

Supplementary Figure 2. (a) Room-temperature cyclic mechanical tests by applying a compressive stress for near-<001> oriented Ti-Al-Cr single crystal, and (b) evolution of σ_{Ms} and σ_{Af} during cyclic tests.

3. <u>Cyclic mechanical tests at room temperature</u>

The mechanical compressive cycling behavior of the Ti–Al–Cr single crystal is shown in Supplementary Fig. 3. With increasing cycle number, the superelastic response evolves from an initially pronounced flag-shaped loop to a more linear stress–strain profile, resembling fatigue behavior commonly seen in shape memory alloys such as NiTi. The transformation stress hysteresis, measured at a fixed strain of 4%, remains nearly unchanged after mechanical training (Supplementary Fig. 3b). After 1600 cycles, the accumulated residual strain is limited to approximately 1.3%, indicating modest functional degradation. Post-cycling EBSD analysis (Supplementary Fig. 3b) reveals the coexistence of B19 martensite and B2 parent phases, confirming that transformation-related microstructural features are preserved even after repeated cycling. These findings suggest that, although minor residual strain and mechanical training effects are present, the stress-induced martensitic transformation remains largely reversible.

Overall, this cyclic stability, both in mechanical response and microstructure, is encouraging for elastocaloric applications, where materials must undergo repeated actuation with minimal performance loss. However, from the viewpoint of long-term device operation, further enhancement in fatigue resistance

remains an important direction for future research. For instance, microstructural optimization through alloy design strategies such as composition tuning or orientation controlling to suppress defect formation, promoting better phase compatibility, and enhancing interface mobility, may help mitigate the accumulation of stabilized martensite and delay fatigue-induced degradation. These approaches could extend the operational lifetime of the material under cyclic loading–unloading, making it more competitive for practical elastocaloric refrigeration systems.

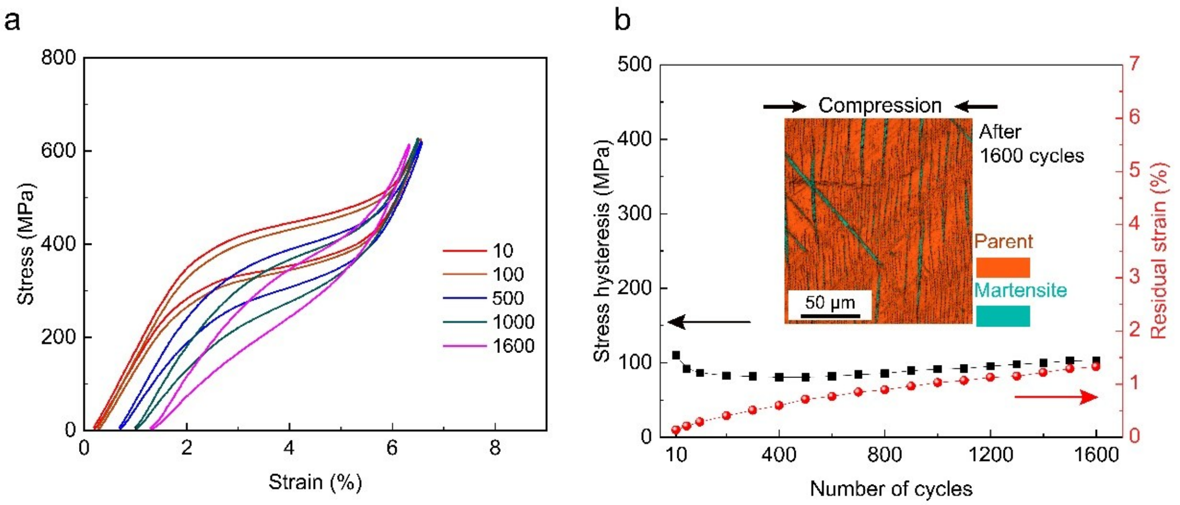

Supplementary Figure 3. (a) Cyclic stress–strain curves of a mechanically trained Ti–Al–Cr single-crystal specimen compressed along a near-[001] orientation, showing the evolution of the superelastic response over 1600 loading–unloading cycles at room temperature. (b) Evolution of residual strain and stress hysteresis as a function of cycle number. The stress hysteresis is defined as the difference between the loading and unloading stresses at a fixed strain of 4%. The recoverable strain remains high (~4%), and the hysteresis width stabilizes after the initial mechanical training cycles. The inset shows the EBSD phase map obtained after cyclic testing, revealing the coexistence of the B2 parent phase and residual B19 martensite.

4. <u>Strain rate dependence of superelastic response</u>

We investigated the strain-rate dependence of the superelastic response in the Ti–Al–Cr single crystal at room temperature. Supplementary Fig. 4 shows the stress–strain curves measured under compressive loading–unloading at three representative strain rates: 0.0005 s^{-1}, 0.05 s^{-1}, and 0.2 s^{-1}. The specimen used

was a mechanically trained near-[001]-oriented single crystal, identical in orientation and thermal–mechanical history to those used in elastocaloric experiments. As shown in Supplementary Fig. 4, all three stress–strain curves exhibit similar characteristics, including transformation start/finish stresses, stress hysteresis, and recoverable strain values. No significant shift in critical stresses or degradation of transformation behavior was observed across more than two orders of magnitude in strain rate.

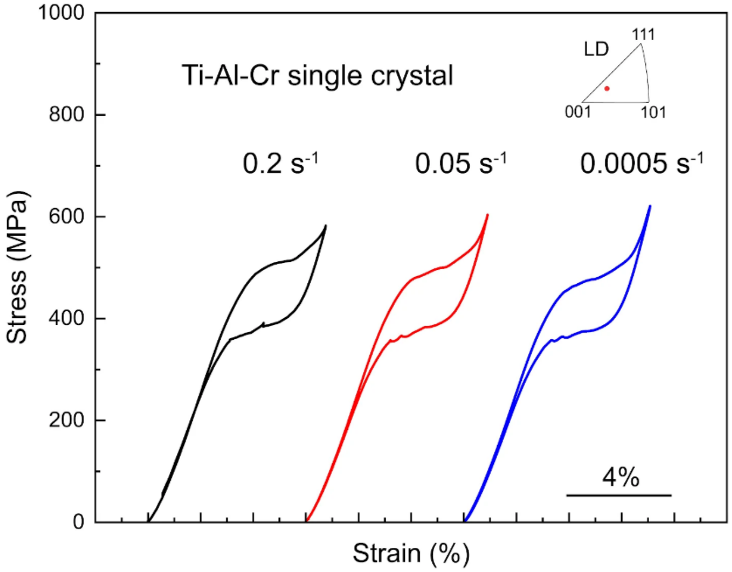

Supplementary Figure 4. Mechanical response of another mechanically trained near-[001]-oriented Ti–Al–Cr single-crystal specimen under compressive loading–unloading at various strain rates at room temperature.

5. <u>Hardening of Young's modulus at cryogenic temperatures</u>

As demonstrated in our earlier work [1], the observed negative temperature dependence of superelasticity in the Ti–Al–Cr alloy is attributed to an increase in elastic modulus with decreasing temperature. As shown in Supplementary Fig. 5, the Young's modulus (E) measured along the compression axis of a near-<001> oriented Ti–Al–Cr single crystal increases sharply below 150 K, particularly in the elastic regime below 200 MPa. This hardening is likely attributed to increased lattice resistance due to shuffle or shear deformation modes towards martensitic transformation, which suppresses transformation kinetics and contributes to the observed negative $d\sigma_0/dT$ anomaly.

Meanwhile, the Cauchy pressure and the bulk-modulus-to-shear-modulus ratio for the cubic crystal can be evaluated from the elastic constants. At room temperature, C_{11} = 129.5 GPa, C_{12} = 101.8 GPa, and C_{44} = 62.8 GPa [1], giving a Cauchy pressure ($C_{12} - C_{44}$) of approximately 39 GPa, characteristic of metallic bonding and consistent with ductile behavior. The bulk modulus is $B = (C_{11} + 3C_{12})/3 \approx 111$ GPa. Using the Voigt–Reuss–Hill averaged shear modulus ($G \approx 35$ GPa), the B/G ratio is approximately 3.2, exceeding the empirical ductility threshold of 1.75.

It is worth noting that cubic single crystals exhibit elastic anisotropy, and the shear modulus depends on deformation mode. In particular, the tetragonal shear modulus $C' = (C_{11} - C_{12})/2 \approx 13.9$ GPa is relatively small, and the elastic anisotropy factor $A = C_{44}/C' \approx 4.5$, reflecting a pronounced soft shear mode associated with lattice instability and facilitating stress-induced martensitic transformation. This elastic softness is consistent with the observed superelastic response.

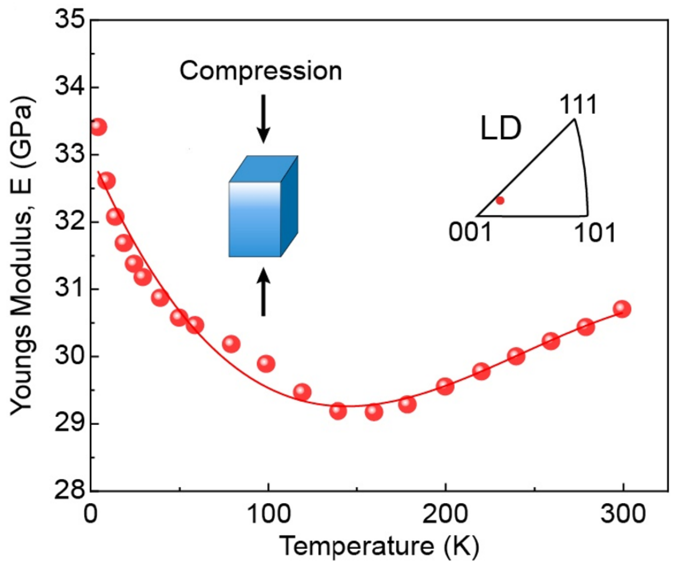

Supplementary Figure 5. Measured temperature dependence of Youngs modulus along loading direction in a single near-<001> oriented Ti-Al-Cr single crystal.

6. <u>Experimental protocol for evaluating eCE</u>

During the measurement of eCE as shown in Supplementary Fig. 6, uniaxial compressive stress is initially applied to the sample. This loading process causes a gradual temperature increase due to the latent

heat released during the stress-induced phase transformation from high-entropy parent phase to low-entropy martensite phase. The load is subsequently maintained for 30 seconds, allowing the sample temperature to return to ambient conditions. Following this stabilization period, rapid unloading is performed at a strain rate of 0.2 s^{-1} to simulate quasi-adiabatic conditions. During this unloading process, the martensite phase reverts to its parent phase, resulting in a significant temperature decrease. The eCE measurements at other temperatures were conducted following the same experimental protocol.

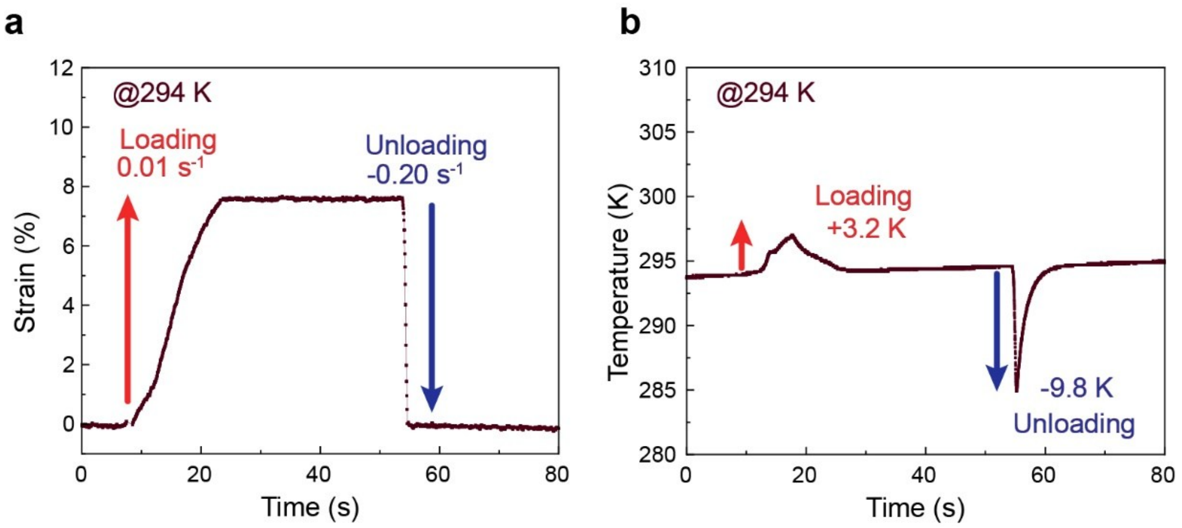

Supplementary Figure 6. (a) Experimental procedures for measuring the adiabatic temperature change during compression tests at 294 K. (b) Temperature increase during loading and decrease during unloading.

7. Thermodynamic evaluation of temperature dependence of superelasticity

The comparison between experimental temperature dependence of superelasticity and thermodynamically calculated temperature dependence of superelasticity was plotted in Supplementary Fig. 7. The thermodynamical calculation below 400 K was based on the reported ΔS as shown in Fig. 3 in the main text. The results show an increasing deviation between experimental values and thermodynamic predictions as temperature decreases, which is probably associated with the hardening in elastic modulus [1].

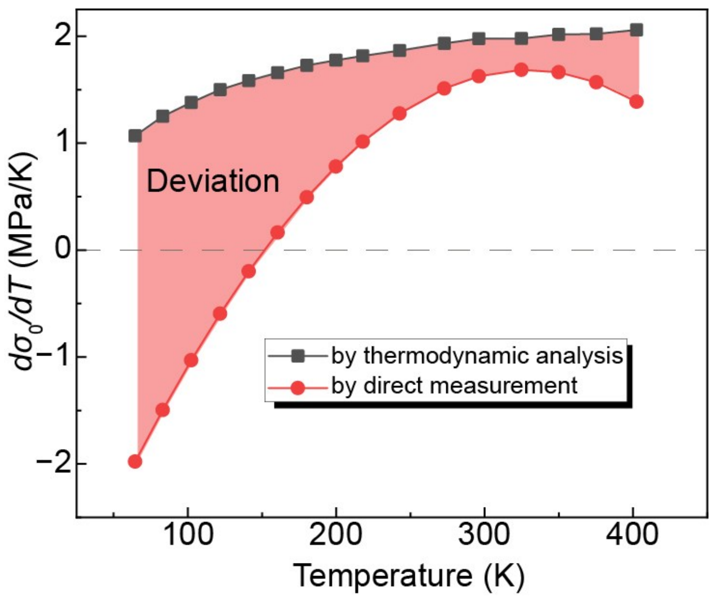

Supplementary Figure 7. Schematic diagrams showing correlation between experimental temperature dependence of superelasticity and thermodynamically calculated temperature dependence of superelasticity using the Clausius–Clapeyron relation.

8. Specific heat estimation at higher temperatures

Fitting of C_p from 300 K to 402 K is reduced from constant volume specific heat C_v by using the following relationship:

$$C_p = C_v + VT\alpha^2 B \quad (1)$$

where V being the molar volume, α being the coefficient of volumetric thermal expansion, and B being the Bulk modulus. Based on previous report, the $V = 1.004 \times 10^{-5}$ m^3/mol and B = 111 GPa for parent phase at room temperature are used [1]. By using $\alpha = 3\alpha_L$, where α_L is the coefficient of linear thermal expansion, the report of a typical β-Ti shape memory alloy yields $\alpha = 2.6 \times 10^{-5}$ /K for parent phase at room temperature [2]. Similarly, B and α of martensite phase are derived from pure titanium [3]. The constant volume specific heat C_v is approximated to heat capacity contributed by the lattice vibration C_{lat} and electronic specific heat C_e which could be expressed by the following equation:

$$C_v = C_{lat} + C_e = 9R\left(\frac{T}{\theta_D}\right)^3 \int_0^{\theta_D/T} \frac{x^4 e^x}{(e^x - 1)^2} dx + \gamma T \quad (2)$$

where R is the gas constant, θ_D is the Debye temperature and γ is the electronic specific heat coefficient. The values of θ_D and γ for both the parent phase and the martensite phase were determined in our previous report [1]. Based on these, the temperature-dependent specific heat C_p was calculated for each phase, and the corresponding entropy change between 300 K and 402 K was evaluated by numerically integrating the fitted C_p curves.

9. Comparison of entropy change (ΔS) derivation methods

In this work, the entropy change associated with the stress-induced martensitic transformation in the Ti–Al–Cr alloy is evaluated using three different approaches, each relying on distinct physical assumptions and experimental conditions. A comparison of these methods is summarized below to clarify their respective scope, advantages, and limitations.

(i) Calorimetric method based on heat capacity measurements

The entropy change is obtained by integrating the difference in specific heat between the B2 parent phase and the B19 martensite phase, which were independently measured using the relaxation calorimetry method. Although no thermally induced martensitic transformation occurs in this alloy, this approach directly reflects the equilibrium thermodynamic entropy difference between the two phases. As such, it is largely free from mechanical dissipation effects and kinetic constraints. This method is therefore considered to provide the most reliable estimate of the intrinsic transformation entropy and represents an upper bound for the achievable elastocaloric temperature change under ideal adiabatic and fully reversible conditions. The effective entropy change for elastocaloric applications should further consider dissipative contributions, including hysteresis loss.

(ii) Clausius–Clapeyron (C–C) relation derived from stress–strain behavior

The entropy change can also be estimated from the temperature dependence of the transformation stress using the Clausius–Clapeyron relation, assuming thermodynamic equilibrium during the stress-induced transformation. This approach is widely used in elastocaloric materials due to its experimental convenience and its direct link to mechanical measurements. In many conventional shape memory alloys, the entropy

changes derived from calorimetry and the C–C relation is comparable. However, in the present Ti–Al–Cr alloy, the temperature dependence of the transformation stress is strongly influenced by anomalous elastic hardening and lattice dynamical effects. As a result, the assumptions underlying the C–C relation are not strictly satisfied, leading to an underestimation of ΔS when this method is applied. This highlights a limitation of the C–C approach in systems where transformation kinetics and lattice dynamics play a significant role beyond classical thermodynamics.

(iii) Back-calculation from experimentally measured ΔT_{ad}

An effective entropy change can be inferred from the directly measured adiabatic temperature change using the relation $\Delta S_{eff} = -C_p \cdot \Delta T_{ad}/T$. This method reflects the actual entropy exchange occurring during a mechanical cycle under realistic experimental conditions. It inherently includes the effects of non-ideal adiabaticity, thermal losses, incomplete transformation, and hysteresis-related dissipation. Consequently, the resulting ΔS represents a lower bound of the transformation entropy and is particularly relevant for assessing practical device-level performance. Its limitation lies in its sensitivity to experimental conditions and measurement techniques, such as strain rate and temperature sensing accuracy.

10. Comparison of COP and efficiency among typical elastocaloric materials

A common metric used to evaluate the performance of elastocaloric materials is the material coefficient of performance (COP_{mater}), defined as the ratio of cooling output (Q) to the mechanical input work (W), assuming fully recoverable unloading energy and no auxiliary power consumption. Here, the cooling output Q is estimated from the measured adiabatic temperature change using $Q \approx C_p|\Delta T_{ad}|$, while the mechanical input work W is taken as the dissipated energy (denoted as Q_{diss} in the main text), evaluated from the hysteresis area of the stress–strain loop. Using the specific heat capacity of the parent phase (588 $J \cdot kg^{-1} \cdot K^{-1}$) [1], the measured adiabatic temperature change (9.8 K), and the hysteretic energy loss derived from the whole stress–strain loop (1260 $J \cdot kg^{-1}$), the COP_{mater} at room temperature (294 K) for the present Ti–Al–Cr alloy is calculated to be 4.6. However, in practical elastocaloric applications, COP_{mater} alone is not a

sufficient metric for comparison, since elastocaloric refrigerants operate between a low-temperature heat source (T_c) and high-temperature heat sink (T_h).

Therefore, the coefficient of performance based on the reverse Stirling cycle ($COP_{stirling}$) is also calculated to provide a more realistic performance metric for comparing different elastocaloric materials operating under a reverse Stirling cycle [4]:

$$\mathrm{COP_{stirling}} = \frac{Q}{(T_\mathrm{h}-T_\mathrm{c})\Delta S+W}. \quad (3)$$

Here, ΔS represents the isothermal entropy change associated with the martensitic transformation, which is assumed to remain approximately constant in the vicinity of ambient temperature. It should be noted that Tc and Th may vary depending on device design and practical environments. Nevertheless, for a consistent comparison among different shape memory alloys, T_c = 283 K and T_h = 293 K are adopted following the reported literature [4].

Under these conditions, the Carnot coefficient of performance for a heat pump operating between Tc and Th, defined as:

$$COP_{carnot} = \frac{T_c}{T_h - T_c}\,, \quad (4)$$

is calculated to be 28.3. The normalized efficiency relative to the Carnot limit, expressed as the ratio of $COP_{stirling}$ to COP_{carnot}, is then evaluated.

The summarized COP or efficiency values of the Ti–Al–Cr alloy and various shape memory alloys are listed in Supplementary Table 1. Although the $COP_{stirling}$ of the present Ti–Al–Cr alloy is comparable to those of commercial NiTi and Cu–Zn–Al shape memory alloys, the $COP_{stirling}$ / COP_{carnot} ratio remains relatively lower than that of nanocrystalline NiTi or TiNiCuCo shape memory alloys. Further improvements, such as composition optimization to increase the isothermal entropy change or reduce hysteretic losses, may enhance the performance of the Ti–Al–Cr alloy for practical applications. It should also be noted that the present comparison is limited to a narrow temperature span near room temperature and therefore does not fully reflect the cryogenic elastocaloric performance of the Ti–Al–Cr alloy.

Supplementary Table 1. Comparison of cooling output, mechanical work, and COP values of representative elastocaloric materials under identical temperature span (T_c = 283 K, T_h = 293 K)

**Material**	$\|Q\|$ (J g^{-1})	$\|W\|$ (J g^{-1})	COP_{mater}	$COP_{stirling}$	COP_{carnot}	$COP_{stirling}$ / COP_{carnot}	**Ref.**
NiTi	9.36	1.78	5.3	4.4	28.3	0.157	[5]
CuZnAl	6.05	1.00	6.1	5.0	28.3	0.176	[6]
NiMnTi	12.74	1.64	7.7	6.1	28.3	0.214	[7]
Nanocrystalline NiTi	16.3	1.34	12.1	8.5	28.3	0.300	[8]
TiNiCuCo	6.43	0.46	14.3	9.5	28.3	0.336	[9]
TiAlCr	5.76	1.26	4.6	3.9	28.3	0.138	This work

Supplementary References:

[1] Y. Song, S. Xu, S. Sato, I. Lee, X. Xu, T. Omori, M. Nagasako, T. Kawasaki, R. Kiyanagi, S. Harjo, W. Gong, T. Grabec, P. Stoklasova, R. Kainuma, A lightweight shape-memory alloy with superior temperature-fluctuation resistance. *Nature* **638**, 965–971 (2025).

[2] M. Bönisch, A. Panigrahi, M. Stoica, M. Calin, E. Ahrens, M. Zehetbauer, W. Skrotzki, J. Eckert, Giant thermal expansion and α-precipitation pathways in Ti-alloys, *Nat. Commun.* **8**, 1429 (2017).

[3] R. Boyer, G. Welsch, E. W. Collings, Materials Properties Handbook: Titanium Alloys, ASM International, Materials Park, OH, 1994.

[4] Qian S., Ling J., Y. Hwang, R. Radermacher, I. Takeuchi, Thermodynamic cycle analysis and numerical modeling of thermoelastic cooling systems, *Int. J. Refrigeration* **56**, 65-80 (2015).

[5] J. Cui, Y. Wu, J. Muehlbauer, Y. Hwang, R. Radermacher, S. Fackler, M. Wuttig, I. Takeuchi, Demonstration of high efficiency elastocaloric cooling with large ΔT using NiTi wires, *Appl. Phys. Lett.* **101**, 073904 (2012).

[6] E. Bonnot, R. Romero, L. Mañosa, E. Vives, and A. Planes, Elastocaloric effect associated with the martensitic transition in shape-memory alloys, *Phy. Rev. Lett.* **100**, 125901 (2008).

[7] G. Zhang, H. Wang, Z, Li, B. Yang, H. Yan, L. Zuo, Giant elastocaloric effect covering a wide temperature region in a directionally solidified $Ni_{50}Mn_{30}Ti_{20}$ alloy, *Scr. Mater.* **237,** 115725 (2023).

[8] H. Chen, F. Xiao, X. Liang, Z. Li, Z. Li, X. Jin, N. Min, T. Fukuda, Improvement of the stability of superelasticity and elastocaloric effect of a Ni-rich Ti-Ni alloy by precipitation and grain refinement, *Scr. Mater.* **162**, 230-234 (2019).

[9] H. Lin, P. Hua, Y. Li, Q. Li, K. Yu, J. Yan, Y. Onuki, Q. Wang, C. Su, G. Zhou, S. Sato, K. Huang, J. Luan, Y. Lee, M. Huang, Y. Yang, Y. Ren, Q. Sun, Ultra-stable and large elastocaloric effect in a nano-precipitated bulk TiNiCuCo shape memory alloy, *Mater. Sci. Eng. A* **949**, 149449 (2025).
